# Revealing Plasma Membrane Nano-Domains with Diffusion Analysis Methods

**DOI:** 10.3390/membranes10110314

**Published:** 2020-10-29

**Authors:** Jakob L. Kure, Camilla B. Andersen, Kim I. Mortensen, Paul W. Wiseman, Eva C. Arnspang

**Affiliations:** 1 SDU Biotechnology, Department of Chemical Engineering, Biotechnology and Environmental Technology, University of Southern Denmark, Campusvej 55, 5230 Odense M, Denmark; jlk@igt.sdu.dk (J.L.K.); caba@igt.sdu.dk (C.B.A.); 2 DTU Health Tech, Department of Health Technology, Technical University of Denmark, Ørsteds Plads, Building 345C, 2800 Kgs. Lyngby, Denmark; kimo@dtu.dk; 3 Department of Chemistry, McGill University, 801 Sherbrooke St. West, Montréal, QC H3A 0B8, Canada; Paul.Wiseman@McGill.ca; 4 Department of Physics, McGill University, 3600 Rue University, Montréal, QC H3A 2T8, Canada

**Keywords:** nano-domain, lipid raft, image correlation spectroscopy, single-particle tracking, fluorescence correlation spectroscopy, diffusion

## Abstract

Nano-domains are sub-light-diffraction-sized heterogeneous areas in the plasma membrane of cells, which are involved in cell signalling and membrane trafficking. Throughout the last thirty years, these nano-domains have been researched extensively and have been the subject of multiple theories and models: the lipid raft theory, the fence model, and the protein oligomerization theory. Strong evidence exists for all of these, and consequently they were combined into a hierarchal model. Measurements of protein and lipid diffusion coefficients and patterns have been instrumental in plasma membrane research and by extension in nano-domain research. This has led to the development of multiple methodologies that can measure diffusion and confinement parameters including single particle tracking, fluorescence correlation spectroscopy, image correlation spectroscopy and fluorescence recovery after photobleaching. Here we review the performance and strengths of these methods in the context of their use in identification and characterization of plasma membrane nano-domains.

## 1. Lipid Membrane Nano-Domains

**Figure 1:**
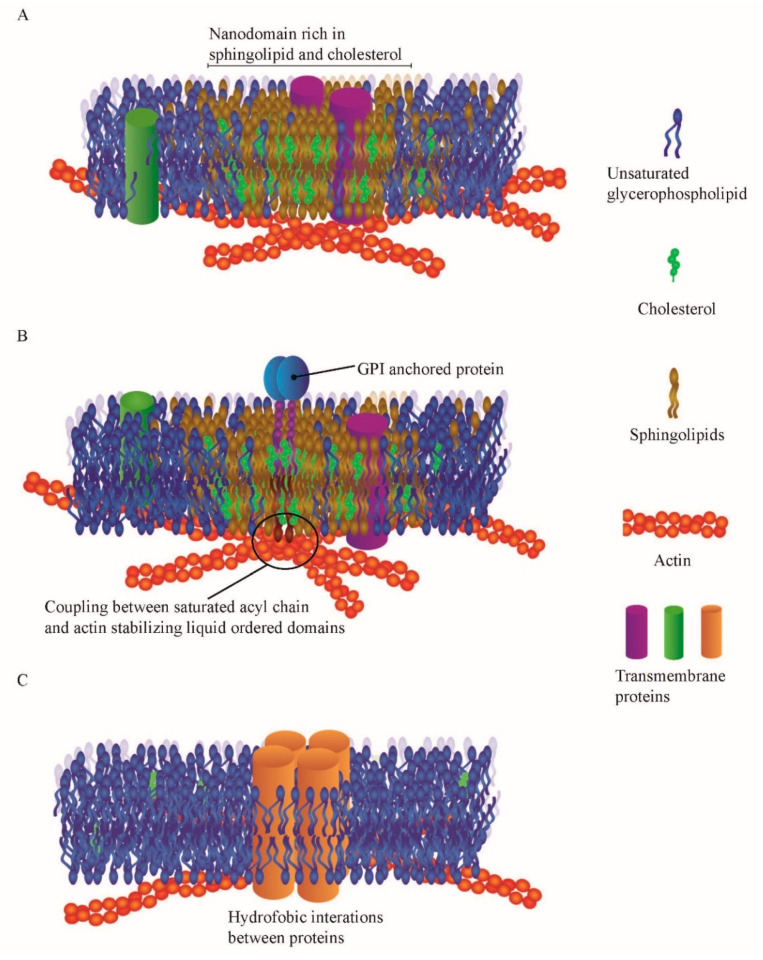
Illustration of the different types of nano-domains in the hierarchal model. (**A**) Illustrates the proteolipid nano-domains, which are stabilized by increased concentrations of sphingolipids and cholesterol. (**B**) Shows the actin anchored nano-domains which are coupled to the underlying actin network. (**C**) Illustrates protein oligomerization that can also appear as nano-domains.

Since the development of the Fluid Mosaic Model [1], multiple new models have been proposed and sensitive methods developed to both explain and probe the heterogeneity of the plasma membrane. After the introduction of the Fluid Mosaic Model, the detergent method supported a central tenet of the Fluid Mosaic Model by providing more evidence of a heterogeneous plasma membrane [2]. The detergent method and modifications of the protocol have been used since then [3,4]. In the 1980s a new very popular description of nano-domains arose from work on model membrane systems, which described the membrane as sub-divided into lipid fractions as liquid disordered and sterol-dependent liquid ordered domains. The liquid ordered fraction, unlike the disordered fraction, is highly structured due to high concentrations of cholesterol ensuring tight packaging that keeps the lipids in place (see Figure 1) [5] The group of model membrane experiments showed, for the first time, that the lipid membrane nano-domains can affect the diffusion of lipids as well as proteins [6]. In the late 1990’s the so-called raft hypothesis was proposed [7] and quickly gained popularity within the field. The raft model describes lipid microdomains enriched with cholesterol and sphingolipids, which can selectively include or exclude various membrane proteins. In 2006 the community agreed upon nano-domains being characterized as small heterogeneous areas in the plasma membrane that are between 10 and 200 nm in diameter, which are enriched with both cholesterol and sphingolipids. Further, they can, over a certain time frame, be stabilized by either protein-protein or protein-lipid interactions [8]. A few years later it was generally accepted that lipid-lipid interactions could also enhance the stabilization of nano-domains [9]. Multiple reviews with a more in-depth history and description of nano-domains are available, see, e.g., [10,11].

With the implementation of higher resolution analytical methods to measure diffusion e.g., single-particle tracking [12], local reductions in the diffusion coefficient and spatial variation in diffusion patterns were observed on cells. It was predicted that these variations were due to nano-domains. However, it gave rise to an alternate hypothesis called the fence model to describe the heterogeneous diffusion behaviour and at the same time questioned the nano-domain theory. The hypothesis was that the diffusive behaviour was mainly affected by the membrane-proximal actin cytoskeleton, which is especially evident in the so-called hop diffusion transport, where the hop is understood to be the protein jumping across the actin grid boundary into an adjacent domain, where it then randomly diffuses around for a period of time, before hopping again into a new actin-filament-defined compartment [13,14]. Later on, the hierarchal model was proposed to describe the plasma membrane [15], which included both the lipid nano-domains and the actin-derived nano-domains but also a group that is defined as oligomerized membrane proteins. The motivation for the hierarchal model is that there is strong evidence that all of these types of domains are present in plasma membranes. The illustrations of the three types of nano-domains can be found in Figure 1.

Nano-domains have been shown to affect multiple mechanisms involved in cell metabolism and function. There is strong evidence for a role of nano-domains in membrane signalling processes [16,17,18] and membrane trafficking [19], moreover, nano-domains are also implicated in various diseases like cancer [20] and cardiovascular diseases [21]. Based on these essential functions of the nano-domains in plasma membrane, it is essential to have valid methods for their identification and characterization to understand their functions in greater detail. This will also give a greater insight into the various nano-domain-derived diseases and possibly aid in evaluating pharmaceutical treatments and ultimately in disease prevention.

Over the time period that models of membrane nano-domains have been developed and refined, multiple analytical methodologies have been introduced to define them through measurements. Many different methodologies are used in current research of nano-domains, including the detergent method, model membranes, cholesterol manipulation, membrane sensitive probing and diffusion methods. The focus of this paper is exclusively to summarize diffusion measurement methods to identify both the size and function of membrane nano-domains. The other methods are well reviewed elsewhere [22,23]. The focus of this review will be to provide insight on the various methods to analyse nano-domains based on molecular diffusive behaviour and will include a discussion about their individual strengths and weaknesses.

## 2. Single Particle Tracking

Single particle tracking (SPT) was implemented for the first time in the 1980s using gold nanoparticles as labels for biomolecules which could be imaged and tracked individually via video microscopy [24]. However, in the mid-1990s, SPT using fluorescent markers was developed, with which tracking of both lipids and receptor proteins in the plasma membrane was demonstrated [25,26]. These application extensions led to a rise in popularity of the method in the early 2000s. This growth has continued over the years and was further augmented with the developments of super-resolution microscopy techniques like photoactivated localization microscopy (PALM) [27] and variants, which have been combined with SPT [28].

**Figure 2:**
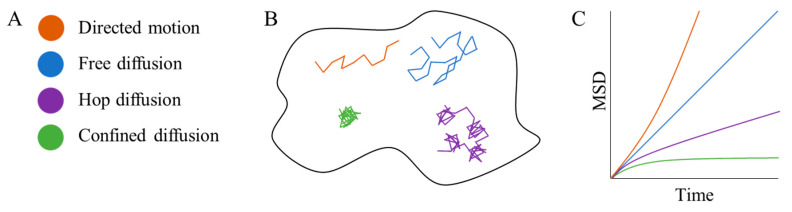
Overview of the most common transport behaviour detected via SPT analysis. The transport modes shown (directed motion, free diffusion, hop diffusion and confined diffusion) have been assigned a color in (**A**). (**B**) Color-coded representative example trajectories for each transport behaviour. (**C**) Schematic of the mean square displacement (MSD) versus time plots calculated from the trajectories for the transport categories described in (**A**,**B**).

SPT follows (i.e., tracks) the movement of imaged labels attached to single particles or molecules, and the resulting trajectories may be analyzed in order to measure the molecular motion. In practice, a molecule is tagged with a fluorophore or alternatively with a more photostable quantum dot nanoparticle, since long tracking periods improve the signal-to-noise ratio for the method [29]. The sample is then imaged as a time-lapse image series (a movie) using a fluorescence microscope. In each frame of such a movie, the position of each biomolecule is detected as an isolated diffraction-limited peak. Each intensity peak is analyzed individually (usually using a 2D Gaussian-model for the intensity distribution) to extract the molecular coordinates with sub-pixel precision (provided that enough photons are recorded per frame). For each molecule, a trajectory may be formed by quantitative linking of the particle coordinates in consecutive frames. The trajectories are then analysed, traditionally via mean squared displacement versus time plots (see below), to reveal the characteristics of the underlying motion. Using the SPT approach, it has been found that the diffusion behaviour of molecules in the cell membrane varies or regularly changes between multiple canonical patterns, namely free diffusion [30], confined diffusion [30], hop diffusion [31], channelled diffusion [32], stimulation-induced temporary arrest of lateral diffusion (STALL diffusion) [33] and directed motion [34]. The most common diffusion types seen are illustrated in Figure 2B. Some of these diffusive behaviours are believed to be the result of nano-domains, where the confined and STALL diffusion is believed to be mainly due to motion and trapping within lipid nano-domains, whereas the hop diffusion is thought to be a result of actin-derived nano-domains.

One key advantage of SPT is that it tracks the motion of *individual* molecules or particles. Consequently, if each trajectory is sufficiently long, no population averaging is needed, and the method may deliver single-molecule results based on single-molecule measurements. This is desirable in the common case where the population of molecules under study shows heterogeneous behaviour in terms of mobility. In order to take advantage of the single-molecule resolution, each trajectory must be analysed individually. Initially, one may identify individual molecules that are immobilized but appear to be moving due to localization errors [35] in the measurement of the molecule’s position [36]. If retained in the downstream analysis, such an immobile fraction of molecules could bias any derived diffusion parameters. Each molecule in the remaining mobile fraction then may and should be analysed individually. If the trajectories are sufficiently long, one can in principle use the mean-squared displacement (MSD) over time to discriminate between free, compartmentalized, and confined diffusion [37], as illustrated in Figure 2C. If trajectories, on the other hand, are short, which is not uncommon when imaging single fluorescent molecules (where photobleaching of the probe limits the acquisition), rigorous classification based on individual trajectories is next to impossible. For instance, when trajectories are short, freely diffusing molecules may be interpreted as diffusing in confinement and vice versa, as demonstrated in simulations [36]. An alternative approach initially assumes normal, free diffusion and proceeds to extract the diffusion coefficient directly and analytically from each trajectory, while accounting for motion blur and localization errors [38,39]. In this case, one should follow up with a statistical test for consistency of trajectories being actual realizations of normal, free diffusion with a diffusion coefficient given by the average measured diffusion coefficients [38,39]. There are two possible outcomes of this: either data are consistent with the hypothesis that the molecules are exhibiting free and normal diffusion and the estimates of diffusion coefficients are reliable, or the data are inconsistent with that hypothesis. In the latter case, one may have to compromise on the single-molecule results and, e.g., obtain the population-averaged MSD. Since free diffusion was already ruled out, it is likely not linear as a function of time-lag. This population-averaged MSD may be consistent with other types of diffusion, however, such as directed, compartmentalized, or confined. If so, diffusion parameters may be extracted by a fit of an appropriate model MSD to the data. This procedure was recently applied to extract diffusion coefficients and sizes of confining regions based on short trajectories obtained in SPT-PALM experiments [36].

One should be wary when using 2D SPT analysis, as it might introduce a bias due to the assumption that the topography plasma membrane is completely flat in the imaged region of the membrane. It has however been shown that this is not completely true, and might actually compromise the diffusion coefficient measurement significantly [40]. It has been proven, that it is possible to do 3D SPT in high resolution [41]. A 3D approach might be a more consistent approach in the identification of diffusion coefficients using SPT in future works.

## 3. Fluorescence Correlation Spectroscopy

Fluorescence correlation spectroscopy (FCS) was originally developed as a tool to identify binding of a fluorescent marker (ethidium bromide) to DNA molecules in a solution [42]. In practice, the technique measures fluctuations in the fluorescence intensity as the number of fluorescent molecules changes in time from within a tiny specific area or volume that is exposed to a tightly focused laser beam. Intensity fluctuations arise from any process that changes the number of fluorescent molecules within the excitation focal spot, e.g., reactions that reduce or increase fluorescence, or molecular transport such as flow and/or diffusion that can either increase or decrease the density of fluorescent particles in the laser beam focus [42]. FCS analysis of the intensity time series entails calculation of a normalized time autocorrelation function of the intensity fluctuations as seen in Equation (1), where *δI* is the amplitude of fluorescence fluctuation and <*I*> is the average fluorescence intensity from the time series, and *τ* is the time lag or shift parameter of the autocorrelation function. The calculated autocorrelation function can be fitted with a decay model and the characteristic fluctuation time is extracted as a fitting parameter if the focal spot size is known. The characteristic fluctuation time is effectively the average residency time for the fluorescent molecule to be present within the illumination spot [42].

(1) $$G\left(\tau \right)=\frac{\langle \delta I\left(t\right)\times \delta I\left(t+\tau \right)\rangle }{\langle I^{2}\rangle }$$

**Figure 3:**
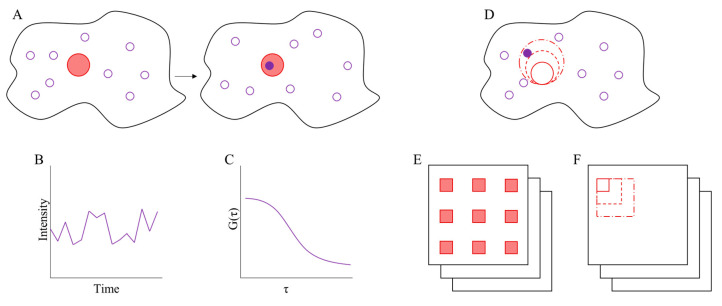
A schematic overview of FCS basics and techniques. (**A**–**C**) shows the basic principle in FCS fluorescence fluctuation analysis. (**A**) Shows that when a fluorescent particle (purple circle) diffuses into the laser focal spot (red circle) on a cell (black circle), it will be excited and emit fluorescence photons which are collected and detected. The random movement of fluorescent molecules in and out of the laser focal spot will result in a fluctuating intensity plot which reflects changes in the number of fluorophores in the focal spot as seen in (**B**). A time auto-correlation function of the fluctuations is calculated and fitted with an appropriate decay function as seen in (**C**). Measurement of the focal spot radius combined with the characteristic fluctuation time from the autocorrelation fit allows calculation of D for the fluorescent molecules. (**D**–**F**) Illustrates the different variants of FCS techniques described in the main text. (**D**) Shows the svFCS where the laser focus area is increased, which is utilized to measure the diffusion coefficient and test for confinement on different spatial scales. (**E**) Illustrates the imFCS method where the fluorescence fluctuation analysis is applied to an image stack over multiple region of interest (ROI), where each red square corresponds to an individual ROI. (**F**) Showcases bimFCS, which is in principle the imaging version of the svFCS.

Shortly after the development of FCS, it was applied with the aim of detecting and measuring the mobility of molecules in cell membranes. Unfortunately, instrumental limitations did not allow such measurements at the time, and needed the technological development provided by scanning FCS (sFCS) in the mid-1980s [43] and confocal pinhole FCS [44]. These later developments significantly accelerated the use of FCS within the biological fields. The scanning method made it possible to move the laser beam around and thereby getting a bigger scanning area, without increasing the time resolution significantly. This made it possible to identify aggregates and slow-moving molecules in cell membranes, and thereby characterizing diffusion and flow behaviour. With the discovery of nano-domains, FCS was a natural tool to apply for their characterization. Further extensions to FCS have been developed, which are able to provide more precise results for nano-domain research including imaging FCS (ImFCS), spot-variation FCS (svFCS) and binned-imaging FCS (bimFCS). An illustration of the different FCS methods is shown in Figure 3. Additionally, FCS is often the go-to technique when designing innovative experimental setups for diffusion analysis; an example of this is the combination of FCS and optical nano-antennas that can be used to identify nano-domains [45,46].

### 3.1. Imaging FCS

A significant extension of FCS is the ImFCS technique [47], which uses a highly sensitive camera, e.g., an electron multiplying charge-coupled device (EMCCD) camera, combined with total-internal reflection fluorescence microscopy (TIRFM) for area- instead of point-detection. The system is able to apply the FCS technique across the pixel array to measure diffusion over entire cells and thereby improving the throughput and the scope of the analysis [48]. The method analyses times series from [44] multiple pixels across the image to determine the FCS measurement for an area, however there should be a certain distance between the pixels, from which the signal is measured, to avoid cross-talk between signals in neighbouring pixels [47].

This tool has been utilized to identify and describe nano-domains in regard to both diffusion coefficient and lateral distribution [49].

### 3.2. Spot Variation FCS

Another method that has become quite popular in the plasma membrane field is the spot variation FCS (svFCS) method [50]. Shortly after its initial introduction, it was applied to identify and characterize membrane nano-domains [51]. The method is based on changing the size of the focal spot area illuminated by the laser beam, which will result in an increase of the transit time of fluorescent biomolecules for increasing spot sizes. The increase follows a linear model, with the slope being the inverse of the effective diffusion coefficient. The intercept can be utilized to identify the diffusion-confinement type in order to classify the category of membrane nano-domain. If the intercept is significantly positive it will be identified as a STALL diffusion type, if the intercept is significantly negative it correlates to hop diffusion representative of actin derived nano-domains, and if the intercept is zero it is described as free diffusion. However, this method still involves extrapolation because the size of the nano-domains is below the light diffraction limit resolution [52]. It required the development of the stimulated emission depletion (STED) microscopy [53] to extend this method further, hence it allowed for analysis with resolutions far below the diffraction limit. An example of svFCS combined with STED microscopy showed a clear differentiation between free diffusion of phosphoethanolamine and STALL-like diffusion of sphingomyelin, supporting the proteo-lipid nano-domain theory [54]. Since then, the STED-FCS combination has become very popular for application within multiple research areas, including plasma membrane research, which has led to commercialization of dedicated STED-FCS instruments.

### 3.3. Binned-Imaging FCS

Binned-imaging FCS (bimFCS) is conceptually similar to svFCS [55]. The difference is mainly in the data acquisition, where the bimFCS is collected as TIRFM images and then analysed. When the image has been acquired, bimFCS utilizes the spot variation technique, which for bimFCS is applied by grouping pixels into different sizes of “super-pixels” e.g., 2 × 2, 3 × 3 and 4 × 4 pixels. After the construction of the required super-pixels, the same data analysis as in svFCS is applied and interpretation and classification of diffusion coefficient and confinement class remain unchanged. This method has proven to discriminate diffusion behaviour and is therefore a great choice for identifying membrane nano-domains if a TIRFM is available [56]. A detailed step by step protocol for bimFCS analysis can be found in [57].

## 4. Image Correlation Spectroscopy

Image correlation spectroscopy (ICS) is a technique developed as an extension to the scanning FCS technique [58]. ICS extended fluorescence correlation spectroscopy with a wide-field view to investigate the spatial domain by using fluorescence microscopy images as input for analysis. The first applications of the method analysed fluorescence images from confocal laser scanning microscopes and employed only spatial image correlation analysis on static samples (chemically fixed cells) to measure cell surface receptor densities and aggregate size distributions [59]. However, over time, the method was extended to different types of fluorescence and super-resolution microscopes and different types of correlation analysis (spatial, temporal, *k*-space and time) which made it possible to extend the range of parameters that could be analysed by ICS analysis.

ICS is thoroughly described in [58] and is a technique that analyses the intensity fluctuations input from fluorescence microscopy images collected as time series from a cellular (or in vitro) sample. An intensity fluctuation is defined as the intensity difference of a pixel from the mean intensity of either an entire image area or a specific region of interest (ROI) (or for temporal ICS the mean intensity of a pixel stack in time) [60].

Since its inception in the early 1990′s, multiple variants of the ICS were developed to optimize different types of measurements. These extensions included temporal image correlation spectroscopy (TICS) [61], image cross-correlation spectroscopy (ICCS) [62], spatio-temporal image correlation spectroscopy (STICS) [63], *k*-space image correlation spectroscopy (kICS) [64], raster-scan image correlation spectroscopy (RICS) [65,66], particle image correlation spectroscopy (PICS) [67] and photobleaching image correlation spectroscopy [68]. Real-space ICS techniques can generally be classified as a subset of the same generalized spatiotemporal correlation function [69] (this does not include reciprocal space kICS [60]):

(2) $$r_{ab}\left(\xi ,\eta ,\tau \right)=\frac{\langle \delta i_{a}\left(x,y,t\right)\times \delta i_{b}{(x+\xi ,y+\eta ,t+\tau )\rangle }_{xy}}{\langle i_{a}{\left(x,y,t\right)}_{t}\rangle \times \langle i_{b}{(x,y,t+\tau )\rangle }_{t+\tau }}$$

The intensity, i(x,y,t), is defined in image space by pixels (*x*,*y*) and in time (*t*) where *t* is the time collection point for each frame in the image series. The fluorescence intensity fluctuation, δi(x,y,t), is defined as:

(3) δi(x,y,t)=i(x,y,t)−〈i(x,y,t)〉

where <*i*(*x*,*y*,*t*)> is the mean intensity average over space for the image or ROI.

The angular brackets in the numerator of Equation (2) indicate the calculation of a spatial correlation function averaged over pairs of images separated by time (frame) lags through the image time series and this is normalized by mean intensities as shown. The calculation is repeated for each sequentially increasing discrete integer step from 0 to the maximum time lag (N image frames). The overall space-time correlation is a function of pixel shift (i.e., spatial lags) variables and frame shift (i.e., temporal lag) variable.

### 4.1. Spatio-Temporal Image Correlation Spectroscopy

Spatio-temporal image correlation spectroscopy (STICS) is an extension of the original ICS methods that analyses imaged fluorescence fluctuations in space and time by calculating the full spatio-temporal correlation function represented by Equation (2) for a single imaging collection channel (i.e., a = b). STICS has proven to be very useful for biomolecule flow measurements in static and motile cells for identifying directed movement of various labelled molecules [63]. STICS has been applied to map transport of component proteins in and out of forming and disassembling membrane mechanosensory podosome complexes in human dendritic cells [70].

The basic STICS method has been extended to mapping co-transport of two different biomolecules labelled with fluorophores of different emission wavelengths and imaged in two different detection channels. This derivative is termed spatio-temporal image cross-correlation spectroscopy (STICCS) and the analysis involves the calculation of the full spatio-temporal correlation function represented by Equation (2) for two imaging collection channels (i.e., a ≠ b) from the dual color image times series collected for the two different labelled molecules.

More recently, an extension has been developed for STICS called velocity landscape correlation (VLC), which is able to extract multiple flow velocities from the extension of the basic STICS analysis [71]. Such extensions, including iMSD (see below), should prove useful for future work in the analysis of nano-domains.

STICS can be used for diffusion measurements and is even able to identify diffusion behaviour in a similar manner to SPT [72]. Compared to SPT, STICS can successfully measure at higher densities of photoactivated molecules and a lower signal/noise ratio. However, as an ensemble method, STICS does not provide single molecule trajectory data, which is one of the greatest advantages of SPT [72].

### 4.2. Imaging Mean Square Displacement

An extension to the STICS method called imaging mean square displacement (iMSD) has also been introduced recently. The iMSD method measures the time-dependent spreading of the width of the space-time correlation function in order to quantify an ensemble averaged MSD as a function of time from the imaged region. The iMSD technique can detect mean displacements between 25 and 100 nm without any a priori assumptions on the diffusion properties of the molecules [73] and can be carried out at multiple scanning speeds [74]. The iMSD approach returns the time-dependent variance of the space-time correlation function by fitting the correlation function with a Gaussian fit as a function of time. A plot of the correlation function variance as a function of time is the averaged MSD(t) curve so the slope, plateau and intercept report similar molecular parameters (diffusion coefficient, confinement type) as SPT but as an ensemble average so the method is rapid [73]. The iMSD has seen use in both intracellular trafficking [75] and plasma membrane dynamics [76]. The work done on plasma membrane dynamics showed that the iMSD measurement can resolve confined, partially confined and linear diffusion of membrane components, which can be used to identify nano-domains in the plasma membrane [76].

### 4.3. PICS

The particle image correlation spectroscopy (PICS) method combines the advantages of SPT and ICS [67], resulting in a technique that functions at a high particle density, but is less impacted by the optical resolution limit compared to non-super-resolution traditional ICS analysis methods. The model is created assuming ideal conditions of the diffusing particles, however, there are modifications to the PICS algorithm, that can be applied if the specific analysis does not meet the assumptions, as would be the case for multiple diffusion coefficients [67].

PICS has been shown to be able to discriminate and measure two diffusion coefficients, which is a great advantage, when working on analysing plasma membrane organization including nano-domains. The PICS model may not be valid if there are protein-protein interactions that do not follow the mean-field approximation [67]. This could be an issue regarding membrane proteins, since these interactions can be very complex especially in consideration of confinement within nano-domains.

### 4.4. k-Space Image Correlation Spectroscopy

The k-space image correlation spectroscopy (kICS) is a reciprocal space variant of ICS and was developed to allow measurements of diffusion and/or flow in the presence of complicating on/off emission blinking of the probes as was exhibited by quantum dot nanoparticles [64]. With kICS, each image in an acquired image stack is Fourier-transformed in space with a 2D FFT to convert to an image time series of spatial frequencies (not spatial pixels). Time correlation is then performed on the spatial frequency image series from the Fourier transform and the output, which is the kICS correlation function, is analysed with appropriate models. If the emission photoblinking is strictly time dependent, the kICS analysis can separate the photoblinking correlations in time from the space-time dependent molecular transport correlations. The kICS method has been shown to successfully measure the dynamics of membrane macromolecules labelled with quantum dot nanoparticles and has also been able to extract both flow velocities and diffusion coefficients that match values from SPT measurements [64]. It has further been proven that kICS, unlike SPT can function both in high and low density labelled macromolecule environments, making the method more flexible than SPT in the molecular density dynamic range [77]. The *k*-space time correlation function is represented symbolically as follows:

(4) $$r\left(k,\tau \right)=\langle \tilde{i}\left(k,t\right)\times {\tilde{i}}^{*}{\left(k,t+\tau \right)\rangle }_{t}$$

where $\tilde{i}$ are the Fourier transformed images, * represents complex conjugation of the Fourier transformed image, and the angular brackets indicate a time autocorrelation. If the point spread function is symmetric, the *k*-space time correlation function is radially averaged and then the natural logarithm is applied. If the photophysics of the fluorophore is only time dependent (not spatial), there is only diffusion (not flow) and there is only one diffusing fluorescently labelled species, then we obtain equation 5 which is used for kICS analysis:

(5) $$\ln\left(r\left({\left|k\right|}^{2},\tau \right)\right)=\ln\left(N\times \frac{q^{2}I_{0}^{2}\omega_{0}^{4}\pi^{2}}{4}\times \langle \theta \left(t\right)\times \theta \left(t+\tau \right)\rangle \;\right)-{\left|k\right|}^{2}\times \left(D_{\tau }+\frac{\omega_{0}^{2}}{4}\;\right)$$

where *N* is the number of particles in the image, *q* is the quantum yield, *I*_0_ is the peak central intensity of the focused laser, *ω*_0_ is the e^−2^ laser beam radius, *θ* is a photophysics variable describing if the particle is fluorescing (1 if yes and 0 if no), and *D_τ_* is the diffusion coefficient.

**Figure 4:**
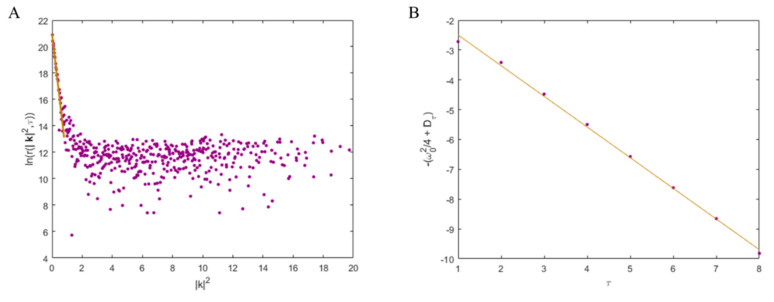
Figures showing kICS correlation function analysis to measure a diffusion coefficient for a labelled molecule in a 2D system. (**A**) An example of a simulated image stack analysed with equation 5 at a given lag time *τ*. The linear fit is shown for |*k*|^2^ between 0 and 0.8 to obtain the diffusion coefficient for k-vector scales still exhibiting correlation above the noise floor for this time lag. This linear fit slope is measured at multiple *τ* lag values and plotted versus lag time as seen in (**B**). The slope of this slope versus time lag plot is fit with a linear regression in (**B**) and the diffusion coefficient is the negative of the fit slope value (in this case 1 µm^2^/s).

A plot of the natural logarithm of the radially averaged *k*-space correlation function versus ${\left|k\right|}^{2}$ will decay linearly for pure diffusion and the *y*-intercept with depend on the emission photophysics correlations, instrumentation factors and the molecular density. The plot is fitted from its linear decay regime through to a noise floor using a three-knotted spline of first order. The initial linearly decreasing part of the spline spans *k*-vectors where molecular transport is still correlated for a given time-lag. The slope of the linear correlation decays is collected for each time lag and plotted against the time lag resulting in another linear plot to which linear regression is applied and the slope is the diffusion coefficient [60,64]. An example of a kICS analysis plot is given in Figure 4.

An extension of kICS analysis was applied to study nano-domains in cell membranes by a two-component model that could detect and measure the motion and fractional distribution of e.g., a protein inside and outside domains smaller than the PSF focus [78,79].

## 5. Fluorescence Recovery after Photobleaching

Fluorescent recovery after photobleaching (FRAP) was a technique developed in the 1970s [80,81] and is still commonly used to measure biomolecule diffusion in cells.

The method works by focusing a laser on a small area of a fluorescently labelled cell, and after a low intensity initial fluorescence baseline measurement in time, the laser is increased to high intensity for a brief time period. The high intensity perturbation photobleaches the fluorophores and, consequently, the illumination area becomes dark as the emitting fluorophores are depleted. The illumination laser is switched back to its lower observation intensity and the fluorescence intensity from the bleached region is monitored over time. The fluorescence signal in the photobleached area will recover over time due to labelled molecules diffusing (or flowing) back into and photobleached molecules diffusing (or flowing) out of the FRAP observation area. After a certain time, the maximal recovery will be reached as a plateau in the intensity recovery curve. If this does not match the initial pre-bleach intensity, it indicates the presence of an immobile fraction of labelled molecules. The recovery curve can be fit with an appropriate model to obtain the diffusion coefficient for the mobile fraction. Various derivatives of FRAP have been developed to improve the technique. Line FRAP is an important extension which can be used in research on membrane nano-domains as it allows for faster and more localized measurements of diffusion coefficients due to a smaller region being bleached [82].

FRAP has been widely applied for studies on membrane nano-domains, by measuring the diffusion coefficients and immobile fractions of nano-domain related proteins. This has been achieved by manipulating the cholesterol levels in the cellular plasma membrane using methyl-β-cyclodextrin (MBCD) and then observing the changes in the diffusion coefficients via FRAP [83]. An increase in diffusion coefficient would mean that there are fewer or smaller nano-domains to slow down molecules resulting in faster recovery, while a measured slower diffusion coefficient would indicate the opposite result. The same scenario could also be explored with an agonist that is expected to activate proteins and “trap” these in nano-domains, which would result in slower FRAP recovery. An agonist approach would probably be more ideal to identify nano-domains and especially their involvement in cell metabolism. The reason for this is that cholesterol manipulation in the plasma membrane can also alter the intracellular cholesterol content and thereby have a huge negative impact on the physiology and metabolism of a living cell [84].

## 6. Discussion

There are many techniques which are able to measure the diffusion coefficients of proteins and lipids in plasma membranes and all of them are widely used in the nano-domain research field. All of these methods have both strengths and weaknesses and the strengths are amplified and weaknesses compensated for when multiple techniques are used to address the same system. The greatest strength of SPT is that it is possible to get trajectories from individual molecules, which makes it possible to visually define the transport behavior of each labelled molecule. However, SPT is much more time consuming as it requires many replicates to obtain statistically meaningful results for the diffusion coefficient and SPT begins to fail when the labelled molecule density is too high. These weaknesses of the SPT can be solved by applying either the FCS or ICS techniques. Among the correlation-based techniques, FCS is the most developed for the identification of nano-domains and in particular the svFCS and bimFCS methods have advantages, as they also provide some information about confinement-induced deviations from free diffusion behavior along with the diffusion coefficients. They are, however, limited to only detecting three kinds of anomalous diffusion behavior, whereas SPT trajectories can reveal all diffusion and transport patterns. The original FCS and imFCS techniques work well for measuring diffusion coefficients and detecting changes in the diffusion, however they do not have the ability to clearly define transport modes that deviate from free diffusion apart from assessing anomalous diffusion. This is also the case for most of the ICS techniques. The development of STICS to work in a similar fashion as SPT but for higher densities (ensembles) of labelled molecules has certain advantages in cell expression systems (e.g., fluorescent proteins) and might be able to be developed further in combination with super-resolution microscopy for the identification and characterization of nano-domains. PICS have also shown some great promise in the identification of nano-domains but the complication of protein-protein interactions might present a significant challenge when investigating the interaction between proteins and nano-domains. The FRAP technique has some of the same strength as FCS and ICS, but when using FRAP it is difficult to sample large representative areas for analysis. However, it still remains an excellent tool to define the diffusion coefficient and also to identify the immobile fraction of the molecule of interest in the plasma membrane.

All the mentioned techniques have added to the current knowledge of plasma membrane organization and nano-domains. This is exemplified by how SPT has helped in identifying different diffusion patterns [30,31,32,33], or how the spectroscopic techniques like ICS and FCS have made it possible to use to determine diffusion coefficients corresponding well with SPT measurements in more populated membranes and with less bright fluorophores [78,79]. The pace of development in the bioimaging field is not slowing down. New variants of super-resolution microscopy and fluorescent labelling strategies are pushing the detection limits and will spur new extensions of the currently available analytical techniques to further resolve the mysteries of membrane nano-scale domains.

## References

[1] Singer S.J., Nicolson G.L. (1972). The Fluid Mosaic Model of the Structure of Cell Membranes. Science.

[2] Yu J., Fischman D.A., Steck T.L. (1973). Selective Solubilization of Proteins and Phospholipids from Red Blood Cell Membranes by Nonionic Detergents. J. Supramol. Cell. Biochem..

[3] Sada R., Kimura H., Fukata Y., Fukata M., Yamamoto H., Kikuchi A. (2019). Dynamic Palmitoylation Controls the Microdomain Localization of the DKK1 Receptors CKAP4 and LRP6. Sci. Signal..

[4] Brown D.A., Rose J.K. (1992). Sorting of GPI-Anchored Proteins to Glycolipid-Enriched Membrane Subdomains during Transport to the Apical Cell Surface. Cell.

[5] Sankaram M.B., Thompson T.E. (1991). Cholesterol-Induced Fluid-Phase Immiscibility in Membranes. Proc. Natl. Acad. Sci. USA.

[6] Kahya N., Scherfeld D., Bacia K., Poolman B., Schwille P. (2003). Probing Lipid Mobility of Raft-Exhibiting Model Membranes by Fluorescence Correlation Spectroscopy. J. Biol. Chem..

[7] Simons K., Ikonen E. (1997). Functional Rafts in Cell Membranes. Nature.

[8] Pike L.J. (2006). Rafts Defined: A Report on the Keystone Symposium on Lipid Rafts and Cell Function. J. Lipid Res..

[9] Simons K., Sampaio J.L. (2011). Membrane Organization and Lipid Rafts. Cold Spring Harb. Perspect. Biol..

[10] Lingwood D., Simons K. (2010). Lipid Rafts as a Membrane-Organizing Principle. Science.

[11] Goñi F.M. (2019). “Rafts”: A Nickname for Putative Transient Nanodomains. Chem. Phys. Lipids.

[12] Qian H., Sheetz M.P., Elson E.L. (1991). Single Particle Tracking. Analysis of Diffusion and Flow in Two-Dimensional Systems. Biophys. J..

[13] Kusumi A., Sako Y. (1996). Cell Surface Organization by the Membrane Skeleton. Curr. Opin. Cell Biol..

[14] Albrecht D., Winterflood C.M., Sadeghi M., Tschager T., Noé F., Ewers H. (2016). Nanoscopic Compartmentalization of Membrane Protein Motion at the Axon Initial Segment. J. Cell Biol..

[15] Kusumi A., Suzuki K.G.N., Kasai R.S., Ritchie K., Fujiwara T.K. (2011). Hierarchical Mesoscale Domain Organization of the Plasma Membrane. Trends Biochem. Sci..

[16] Adebiyi A., Soni H., John T.A., Yang F. (2014). Lipid Rafts Are Required for Signal Transduction by Angiotensin II Receptor Type 1 in Neonatal Glomerular Mesangial Cells. Exp. Cell Res..

[17] Varshney P., Yadav V., Saini N. (2016). Lipid Rafts in Immune Signalling: Current Progress and Future Perspective. Immunology.

[18] Kalappurakkal J.M., Anilkumar A.A., Patra C., van Zanten T.S., Sheetz M.P., Mayor S. (2019). Integrin Mechano-Chemical Signaling Generates Plasma Membrane Nanodomains That Promote Cell Spreading. Cell.

[19] Helms J.B., Zurzolo C. (2004). Lipids as Targeting Signals: Lipid Rafts and Intracellular Trafficking. Traffic.

[20] Raghu H., Sodadasu P.K., Malla R.R., Gondi C.S., Estes N., Rao J.S. (2010). Localization of UPAR and MMP-9 in Lipid Rafts Is Critical for Migration, Invasion and Angiogenesis in Human Breast Cancer Cells. BMC Cancer.

[21] Rios F.J.O., Ferracini M., Pecenin M., Koga M.M., Wang Y., Ketelhuth D.F.J., Jancar S. (2013). Uptake of OxLDL and IL-10 Production by Macrophages Requires PAFR and CD36 Recruitment into the Same Lipid Rafts. PLoS ONE.

[22] Sezgin E., Levental I., Mayor S., Eggeling C. (2017). The Mystery of Membrane Organization: Composition, Regulation and Roles of Lipid Rafts. Nat. Rev. Mol. Cell Biol..

[23] Ashrafzadeh P., Parmryd I. (2015). Methods Applicable to Membrane Nanodomain Studies?. Essays Biochem..

[24] Geerts H., De Brabander M., Nuydens R., Geuens S., Moeremans M., De Mey J., Hollenbeck P. (1987). Nanovid Tracking: A New Automatic Method for the Study of Mobility in Living Cells Based on Colloidal Gold and Video Microscopy. Biophys. J..

[25] Schindler H., Feher G. (1996). Imaging of Single Molecule Diffusion. Proc. Natl. Acad. Sci. USA.

[26] Schütz G.J., Kada G., Pastushenko V.P., Schindler H. (2000). Properties of Lipid Microdomains in a Muscle Cell Membrane Visualized by Single Molecule Microscopy. EMBO J..

[27] Betzig E., Patterson G.H., Sougrat R., Lindwasser O.W., Olenych S., Bonifacino J.S., Davidson M.W., Lippincott-Schwartz J., Hess H.F. (2006). Imaging Intracellular Fluorescent Proteins at Nanometer Resolution. Science.

[28] Manley S., Gillette J.M., Patterson G.H., Shroff H., Hess H.F., Betzig E., Lippincott-Schwartz J. (2008). High-Density Mapping of Single-Molecule Trajectories with Photoactivated Localization Microscopy. Nat. Methods.

[29] Clausen M.P., Arnspang E.C., Ballou B., Bear J.E., Lagerholm B.C. (2014). Simultaneous Multi-Species Tracking in Live Cells with Quantum Dot Conjugates. PLoS ONE.

[30] Kusumi A., Sako Y., Yamamoto M. (1993). Confined Lateral Diffusion of Membrane Receptors. Biophys. J..

[31] Fujiwara T., Ritchie K., Murakoshi H., Jacobson K., Kusumi A. (2002). Phospholipids Undergo Hop Diffusion in Compartmentalized Cell Membrane. J. Cell Biol..

[32] Jaqaman K., Kuwata H., Touret N., Collins R., Trimble W.S., Danuser G., Grinstein S. (2011). Cytoskeletal Control of CD36 Diffusion Promotes Its Receptor and Signaling Function. Cell.

[33] Suzuki K.G.N., Fujiwara T.K., Edidin M., Kusumi A. (2007). Dynamic Recruitment of Phospholipase Cγ at Transiently Immobilized GPI-Anchored Receptor Clusters Induces IP_3_-Ca^2+^ Signaling: Single-Molecule Tracking Study 2. J. Cell Biol..

[34] Liu P., Weinreb V., Ridilla M., Betts L., Patel P., De Silva A.M., Thompson N.L., Jacobson K. (2017). Rapid, Directed Transport of DC-SIGN Clusters in the Plasma Membrane. Sci. Adv..

[35] Mortensen K.I., Churchman L.S., Spudich J.A., Flyvbjerg H. (2010). Optimized Localization Analysis for Single-Molecule Tracking and Super-Resolution Microscopy. Nat. Methods.

[36] Arnspang E.C., Sengupta P., Mortensen K.I., Jensen H.H., Hahn U., Jensen E.B.V., Lippincott-Schwartz J., Nejsum L.N. (2019). Regulation of Plasma Membrane Nanodomains of the Water Channel Aquaporin-3 Revealed by Fixed and Live Photoactivated Localization Microscopy. Nano Lett..

[37] Clausen M.P., Lagerholm B.C. (2013). Visualization of Plasma Membrane Compartmentalization by High-Speed Quantum Dot Tracking. Nano Lett..

[38] Vestergaard C.L., Blainey P.C., Flyvbjerg H. (2014). Optimal Estimation of Diffusion Coefficients from Single-Particle Trajectories. Phys. Rev. E.

[39] Vestergaard C.L., Pedersen J.N., Mortensen K.I., Flyvbjerg H. (2015). Estimation of Motility Parameters from Trajectory Data: A Condensate of Our Recent Results. Eur. Phys. J. Spec. Top..

[40] Adler J., Shevchuk A.I., Novak P., Korchev Y.E., Parmryd I. (2010). Plasma Membrane Topography and Interpretation of Single-Particle Tracks. Nat. Methods.

[41] Ram S., Prabhat P., Chao J., Sally Ward E., Ober R.J. (2008). High Accuracy 3D Quantum Dot Tracking with Multifocal Plane Microscopy for the Study of Fast Intracellular Dynamics in Live Cells. Biophys. J..

[42] Magde D., Elson E., Webb W.W. (1972). Thermodynamic Fluctuations in a Reacting System—Measurement by Fluorescence Correlation Spectroscopy. Phys. Rev. Lett..

[43] Petersen N.O. (1986). Scanning Fluorescence Correlation Spectroscopy. I. Theory and Simulation of Aggregation Measurements. Biophys. J..

[44] Rigler R., Mets Ü., Widengren J., Kask P. (1993). Fluorescence Correlation Spectroscopy with High Count Rate and Low Background: Analysis of Translational Diffusion. Eur. Biophys. J..

[45] Winkler P.M., Regmi R., Flauraud V., Brugger J., Rigneault H., Wenger J., García-Parajo M.F. (2018). Optical Antenna-Based Fluorescence Correlation Spectroscopy to Probe the Nanoscale Dynamics of Biological Membranes. J. Phys. Chem. Lett..

[46] Regmi R., Winkler P.M., Flauraud V., Borgman K.J.E., Manzo C., Brugger J., Rigneault H., Wenger J., García-Parajo M.F. (2017). Planar Optical Nanoantennas Resolve Cholesterol-Dependent Nanoscale Heterogeneities in the Plasma Membrane of Living Cells. Nano Lett..

[47] Kannan B., Guo L., Sudhaharan T., Ahmed S., Maruyama I., Wohland T. (2007). Spatially Resolved Total Internal Reflection Fluorescence Correlation Microscopy Using an Electron Multiplying Charge-Coupled Device Camera. Anal. Chem..

[48] Krieger J.W., Singh A.P., Bag N., Garbe C.S., Saunders T.E., Langowski J., Wohland T. (2015). Imaging Fluorescence (Cross-) Correlation Spectroscopy in Live Cells and Organisms. Nat. Protoc..

[49] Bag N., Holowka D.A., Baird B.A. (2020). Imaging FCS Delineates Subtle Heterogeneity in Plasma Membranes of Resting Mast Cells. Mol. Biol. Cell.

[50] Wawrezinieck L., Rigneault H., Marguet D., Lenne P.F. (2005). Fluorescence Correlation Spectroscopy Diffusion Laws to Probe the Submicron Cell Membrane Organization. Biophys. J..

[51] Lenne P.F., Wawrezinieck L., Conchonaud F., Wurtz O., Boned A., Guo X.J., Rigneault H., He H.T., Marguet D. (2006). Dynamic Molecular Confinement in the Plasma Membrane by Microdomains and the Cytoskeleton Meshwork. EMBO J..

[52] He H.-T., Marguet D. (2011). Detecting Nanodomains in Living Cell Membrane by Fluorescence Correlation Spectroscopy. Annu. Rev. Phys. Chem..

[53] Hell S.W., Wichmann J. (1994). Breaking the Diffraction Resolution Limit by Stimulated Emission: Stimulated-Emission-Depletion Fluorescence Microscopy. Opt. Lett..

[54] Eggeling C., Ringemann C., Medda R., Schwarzmann G., Sandhoff K., Polyakova S., Belov V.N., Hein B., Von Middendorff C., Schönle A. (2009). Direct Observation of the Nanoscale Dynamics of Membrane Lipids in a Living Cell. Nature.

[55] Huang H., Pralle A. (2011). Continuous Monitoring of Membrane Protein Micro- Domain Association during Cell Signaling. arXiv.

[56] Huang H., Simsek M.F., Jin W., Pralle A. (2015). Effect of Receptor Dimerization on Membrane Lipid Raft Structure Continuously Quantified on Single Cells by Camera Based Fluorescence Correlation Spectroscopy. PLoS ONE.

[57] Jin W., Simsek M.F., Pralle A. (2018). Quantifying Spatial and Temporal Variations of the Cell Membrane Ultra-Structure by BimFCS. Methods.

[58] Petersen N.O., Höddelius P.L., Wiseman P.W., Seger O., Magnusson K.E. (1993). Quantitation of Membrane Receptor Distributions by Image Correlation Spectroscopy: Concept and Application. Biophys. J..

[59] Wiseman P.W., Petersen N.O. (1999). Image Correlation Spectroscopy. II. Optimization for Ultrasensitive Detection of Preexisting Platelet-Derived Growth Factor-β Receptor Oligomers on Intact Cells. Biophys. J..

[60] Wiseman P.W. (2012). Image Correlation Spectroscopy. Compr. Biophys..

[61] Srivastava M., Petersen N.O. (1996). Image Cross-Correlation Spectroscopy: A New Experimental Biophysical Approach to Measurement of Slow Diffusion of Fluorescent Molecules. Methods Cell Sci..

[62] Wiseman P.W., Squier J.A., Ellisman M.H., Wilson K.R. (2000). Two-Photo Image Correlation Spectroscopy and Image Cross-Correlation Spectroscopy. J. Microsc..

[63] Hebert B., Costantino S., Wiseman P.W. (2005). Spatiotemporal Image Correlation Spectroscopy (STICS) Theory, Verification, and Application to Protein Velocity Mapping in Living CHO Cells. Biophys. J..

[64] Kolin D.L., Ronis D., Wiseman P.W. (2006). K-Space Image Correlation Spectroscopy: A Method for Accurate Transport Measurements Independent of Fluorophore Photophysics. Biophys. J..

[65] Digman M.A., Sengupta P., Wiseman P.W., Brown C.M., Horwitz A.R., Gratton E. (2005). Fluctuation Correlation Spectroscopy with a Laser-Scanning Microscope: Exploiting the Hidden Time Structure. Biophys. J..

[66] Digman M.A., Brown C.M., Sengupta P., Wiseman P.W., Horwitz A.R., Gratton E. (2005). Measuring Fast Dynamics in Solutions and Cells with a Laser Scanning Microscope. Biophys. J..

[67] Semrau S., Schmidt T. (2007). Particle Image Correlation Spectroscopy (PICS): Retrieving Nanometer-Scale Correlations from High-Density Single-Molecule Position Data. Biophys. J..

[68] Ciccotosto G.D., Kozer N., Chow T.T.Y., Chon J.W.M., Clayton A.H.A. (2013). Aggregation Distributions on Cells Determined by Photobleaching Image Correlation Spectroscopy. Biophys. J..

[69] Kolin D.L., Wiseman P.W. (2007). Advances in Image Correlation Spectroscopy: Measuring Number Densities, Aggregation States, and Dynamics of Fluorescently Labeled Macromolecules in Cells. Cell Biochem. Biophys..

[70] Meddens M.B.M., Pandzic E., Slotman J.A., Guillet D., Joosten B., Mennens S., Paardekooper L.M., Houtsmuller A.B., Van Den Dries K., Wiseman P.W. (2016). Actomyosin-Dependent Dynamic Spatial Patterns of Cytoskeletal Components Drive Mesoscale Podosome Organization. Nat. Commun..

[71] Pandžić E., Abu-Arish A., Whan R.M., Hanrahan J.W., Wiseman P.W. (2018). Velocity Landscape Correlation Resolves Multiple Flowing Protein Populations from Fluorescence Image Time Series. Methods.

[72] Pandžić E., Rossy J., Gaus K. (2015). Tracking Molecular Dynamics without Tracking: Image Correlation of Photo-Activation Microscopy. Methods Appl. Fluoresc..

[73] Di Rienzo C., Piazza V., Gratton E., Beltram F., Cardarelli F. (2014). Probing Short-Range Protein Brownian Motion in the Cytoplasm of Living Cells. Nat. Commun..

[74] Gröner N., Capoulade J., Cremer C., Wachsmuth M. (2010). Measuring and Imaging Diffusion with Multiple Scan Speed Image Correlation Spectroscopy. Opt. Express.

[75] Digiacomo L., Digman M.A., Gratton E., Caracciolo G. (2016). Development of an Image Mean Square Displacement (IMSD)-Based Method as a Novel Approach to Study the Intracellular Trafficking of Nanoparticles. Acta Biomater..

[76] Bohórquez-Hernández A., Gratton E., Pacheco J., Asanov A., Vaca L. (2017). Cholesterol Modulates the Cellular Localization of Orai1 Channels and Its Disposition among Membrane Domains. Biochim. Biophys. Acta Mol. Cell Biol. Lipids.

[77] Arnspang E.C., Schwartzentruber J., Clausen M.P., Wiseman P.W., Lagerholm B.C. (2013). Bridging the Gap between Single Molecule and Ensemble Methods for Measuring Lateral Dynamics in the Plasma Membrane. PLoS ONE.

[78] Arnspang E.C., Login F.H., Koffman J.S., Sengupta P., Nejsum L.N. (2016). AQP2 Plasma Membrane Diffusion Is Altered by the Degree of AQP2-S256 Phosphorylation. Int. J. Mol. Sci..

[79] Abu-Arish A., Pandzic E., Goepp J., Matthes E., Hanrahan J.W., Wiseman P.W. (2015). Cholesterol Modulates CFTR Confinement in the Plasma Membrane of Primary Epithelial Cells. Biophys. J..

[80] Peters R., Peters J., Tews K.H., Bähr W. (1974). A Microfluorimetric Study of Translational Diffusion in Erythrocyte Membranes. Biochim. Biophys. Acta Biomembr..

[81] Axelrod D., Koppel D.E., Schlessinger J., Elson E., Webb W.W. (1976). Mobility Measurement by Analysis of Fluorescence Photobleaching Recovery Kinetics. Biophys. J..

[82] Braeckmans K., Remaut K., Vandenbroucke R.E., Lucas B., De Smedt S.C., Demeester J. (2007). Line FRAP with the Confocal Laser Scanning Microscope for Diffusion Measurements in Small Regions of 3-D Samples. Biophys. J..

[83] Oyola-Cintrón J., Caballero-Rivera D., Ballester L., Baéz-Pagán C.A., Martínez H.L., Vélez-Arroyo K.P., Quesada O., Lasalde-Dominicci J.A. (2015). Lateral Diffusion, Function, and Expression of the Slow Channel Congenital Myasthenia Syndrome AC418W Nicotinic Receptor Mutation with Changes in Lipid Raft Components. J. Biol. Chem..

[84] Zidovetzki R., Levitan I. (2007). Use of Cyclodextrins to Manipulate Plasma Membrane Cholesterol Content: Evidence, Misconceptions and Control Strategies. Biochim. Biophys. Acta Biomembr..

